# Prevalence of Nontuberculous Mycobacteria Infection, China, 2004–2009

**DOI:** 10.3201/eid1803.110175

**Published:** 2012-03

**Authors:** Hui Jing, Haiying Wang, Yan Wang, Yunfeng Deng, Xinxin Li, Zhimin Liu, Edward A. Graviss, Xin Ma

**Affiliations:** Shandong Provincial Chest Hospital, Jinan, People’s Republic of China (H. Jing, H. Wang, Y. Wang, Y. Deng, X. Li, Z. Liu, X. Ma);; The Methodist Hospital Research Institute, Houston, Texas, USA (E.A. Graviss, X. Ma)

**Keywords:** nontuberculous mycobacteria, tuberculosis, China, surveillance, population-based, tuberculosis and other mycobacteria

**To the Editor:** Pulmonary nontuberculous mycobacteria (NTM) diseases share clinical signs with tuberculosis (TB), causing a clinical dilemma with regard to therapy for patients with these diseases (*1*). In the past 30 years (post-AIDS era), NTM have increasingly been associated with pulmonary diseases in humans (*2*). Recent studies in urban areas of the People’s Republic of China have shown that the prevalence of NTM (isolation rate of NTM among all mycobacteria) is increasing; for example, prevalence in Shanghai increased from 4.26% in 2005 to 6.38% in 2008 (*3*). To investigate NTM prevalence in rural areas of China, we evaluated the NTM isolation rates, species distribution, and drug-resistance profiles through a population-based TB sentinel surveillance study in Shandong Province, the second largest province in China. The study protocol was approved by the Institutional Review Board of Shandong Provincial Chest Hospital (Jinan, Shandong, China).

Clinical samples were collected through the ongoing sentinel TB surveillance project, which first began in 7 counties in Shandong Province in 2004 and expanded to 13 counties in 2008. Of the total surveillance population, rural populations accounted for ≈80%. Each sample collected in this study was identified only by a unique participant number. Each surveillance site sent sputum samples from all patients with suspected TB to the TB Reference Laboratory of Shandong Provincial Chest Hospital for mycobacterial culture, drug-susceptibility testing, and species identification.

From January 1, 2004, through December 31, 2009, *Mycobacteria* spp. were isolated from sputum specimens from 3,949 patients with suspected pulmonary TB. Of these patients, mean age ± SD was 48.7 ± 20.4 years (range 1–92 years), 74.6% were male, and 300 were being re-treated for TB.

Identification of *Mycobacteria* spp. was first conducted by conventional biochemical testing—p-nitrobenzoic acid and 2-thiophene carboxylic acid hydrazide testing—following a standard protocol (*4*). *Mycobacteria* spp. were further identified by 16S rRNA gene sequence analysis (MicroSeq ID Microbial Indentification Software, version 2.0; Applied Biosystems, Foster City, CA, USA) to the species level as described (*5*). Drug-susceptibility testing was performed according to standard procedures recommended by the World Health Organization, and quality control was conducted by inter-laboratory confirmation testing by reference laboratories recognized by the World Health Organization in South Korea and in Hong Kong Special Administrative Region, China (*6**,**7*). The drug panel included 4 first-line anti-TB drugs: isoniazid, rifampin, streptomycin, and ethambutol.

The conventional biochemical testing of the 3,949 *Mycobacteria* spp. strains identified 68 NTM strains, among which the 16s rRNA gene sequence analysis confirmed 64 (1.6%) NTM strains and identified 3 *M. tuberculosis* complex strains and 1 *Nocardia glanders* strain. Among the 64 NTM strains, 52 (81.2%) were *M. intracellulare*, 5 (7.8%) were *M. kansasii*, 3 (4.7%) were *M. fortuitum*, 2 (3.1%) were *M. chelonae*, 1 (1.6%) was *M. gordonae*, and 1 (1.6%) was *M. scrofulaceum*. The first-line anti-TB drug resistance rates of the 64 NTM strains were 100% for isoniazid, 98.4% for streptomycin, 78.1% for rifampin, and 51.6% for ethambutol (Table). Among the 3,949 *Mycobacteria* spp. strains, 163 (4.1%) were resistant to at least isoniazid and rifampin, of which 50 (30.7%) strains were identified as NTM. Among 300 TB re-treatment cases, 12 (4.0%) were caused by clinically significant NTM infections. Over the 6 study years, NTM isolation rates among the study population did not show a substantial increasing or decreasing trend.

**Table:** Species and drug-resistance profiles of 64 nontuberculous mycobacteria strains, Shandong Province, People’s Republic of China, 2004–2009

*Mycobacterium* species	Resistant strains, no. (%)				
	Total	Isoniazid	Rifampin	Ethambutol	Streptomycin
*M. intracellulare*	52 (81.2)	52 (100)	40 (76.9)	27 (51.9)	51 (98.1)
*M. kansasii*	5 (7.8)	5 (100)	3 (60.0)	0	5 (100)
*M. fortuitum*	3 (4.7)	3 (100)	3 (100)	2 (66.7)	3 (100)
*M. chelonae*	2 (3.1)	2 (100)	2 (100)	2 (100)	2 (100)
*M. gordonae*	1 (1.6)	1 (100)	1 (100)	1 (100)	1 (100)
*M. scrofulaceum*	1 (1.6)	1 (100)	0	1 (100)	1 (100)
Total	64 (100)	64 (100)	50 (78.1)	33 (51.6)	63 (98.4)

Our data suggest that the NTM isolation rate among patients with suspected pulmonary TB in rural China (1.6%) is relatively lower and more stable than that for urban areas (mean rate 5.09% in Shanghai) and that the *Mycobacterium* spp. differ from those in other areas of China (*3**,**8**–**10*). In China and most other developing countries to which TB is endemic, the decision to initiate pulmonary TB treatment is based only on finding a positive sputum smear by microscopy examination, not on *Mycobacteria* culture, species identification, and drug-resistance testing results. Among our study population, NTM strains showed high drug resistance to first-line anti-TB drugs and accounted for 30.7% of suspected multidrug-resistant TB (MDR-TB) cases and 4.0% of TB re-treatment cases.

These findings suggest that pulmonary NTM infections pose substantial difficulties with regard to clinical management of NTM and MDR-TB diseases in China. Laboratory species identification is imperative before proper treatment can be determined for patients with MDR-TB. Compared with conventional biochemical testing, 16S rRNA gene sequencing analysis can more accurately identify *Mycobacteria* spp.
